# A high-sensitivity HPLC assay for measuring intracellular Na^+^ and K^+^ and its application to *Plasmodium falciparum* infected erythrocytes

**DOI:** 10.1038/srep29241

**Published:** 2016-07-07

**Authors:** Markus Winterberg, Kiaran Kirk

**Affiliations:** 1 Research School of Biology, Australian National University, Canberra, ACT 2601, Australia; 2 Centre for Tropical Medicine and Global Health, Nuffield Department of Medicine, University of Oxford, UK.; 3 Mahidol-Oxford Tropical Medicine Research Unit, Faculty of Tropical Medicine, Mahidol University, Bangkok, Thailand

## Abstract

The measurement of intracellular ion concentrations, and the screening of chemical agents to identify molecules targeting ion transport, has traditionally involved low-throughput techniques. Here we present a novel HPLC method that allows the rapid, high-sensitivity measurement of cell Na^+^ and K^+^ content, demonstrating its utility by monitoring the ionic changes induced in the intracellular malaria parasite by the new spiroindolone antimalarial KAE609.

Ion transporters and channels have long been of interest as chemotherapeutic targets for a range of disorders. The cardiac glycosides, used for centuries to treat congestive heart failure, exert their cardioprotective effect by inhibiting the cardiac Na^+^, K^+^ pump^1^. The sulfonylureas, and related drugs, exert their anti-diabetic effect by inhibiting K^+^ channels in the insulin-secreting pancreatic beta cells^2^. Na^+^ channels are the target of local anaesthetics and Na^+^ channel blockers and Ca^2+^ channel blockers are used in the treatment of epilepsy, chronic pain, and cardiovascular disorders^3^. The anti-diarrhoea drug crofelemer exerts its effect by inhibiting Cl^-^ channels in intestinal cells^4^.

Although membrane transport inhibitors have found less application in the treatment of infectious diseases, the recent finding that the new generation ‘spiroindolone’ antimalarials^5^,^6^ disrupt Na^+^ homeostasis in the intraerythrocytic malaria parasite^7^ has highlighted the vulnerability of the malaria parasite’s ion transport mechanisms to chemotherapeutic attack. The disruption of parasite Na^+^ homeostasis is proposed to occur as a result of inhibition of a putative Na^+^-efflux ATPase on the parasite plasma membrane^7^, resulting in an increase in cytosolic Na^+^. The recent revelation that multiple other potential antimalarials^8^,^9^,^10^,^11^, including three designated preclinical drug candidates [http://www.mmv.org/newsroom/news/three-new-roads-leading-common-pathway], exert a similar effect to the spiroindolones on parasite Na^+^ homeostasis has highlighted the potential of the parasite’s putative Na^+^ pump as an ‘Achilles heel’.

Screening compounds for their ability to inhibit cell ion transport mechanisms and thereby disrupt transmembrane electrochemical gradients has generally involved low- to medium-throughput methodologies based on such techniques as flame photometry or atomic absorption spectrometry^12^,^13^, fluorescent ion-sensitive dyes^7^,^8^,^9^,^10^,^11^, or ion-sensitive electrodes^14^ or microelectrodes^15^. These methods are typically limited to the analysis of single ions and/or require large sample volumes/cell numbers and/or complex sample preparation or experimental manoeuvres.

Here we describe a simple, rapid, high-sensitivity method that allows the quantitation of cell Na^+^ and K^+^ from a single trace derived from a single low volume (<10 μL) cell extract, prepared from as few as 10^5^ cells. The method entails washing then lysing a small aliquot of cells, with the resulting extract then analysed by HPLC, using a Charged Aerosol Detector to detect and quantify Na^+^ and K^+^. The assay is readily adapted to a 96-well plate format. It can be used to determine the Na^+^/K^+^ content of any cell type and may be extended to include other common ions. Here we demonstrate the utility of the method by quantifying Na^+^ and K^+^ in the host and parasite compartments of the malaria-parasite-infected human erythrocyte, and by demonstrating the disruption of parasite ion homeostasis by the clinical candidate spiroindolone KAE609.

## Results and Discussion

**Figure 1:**
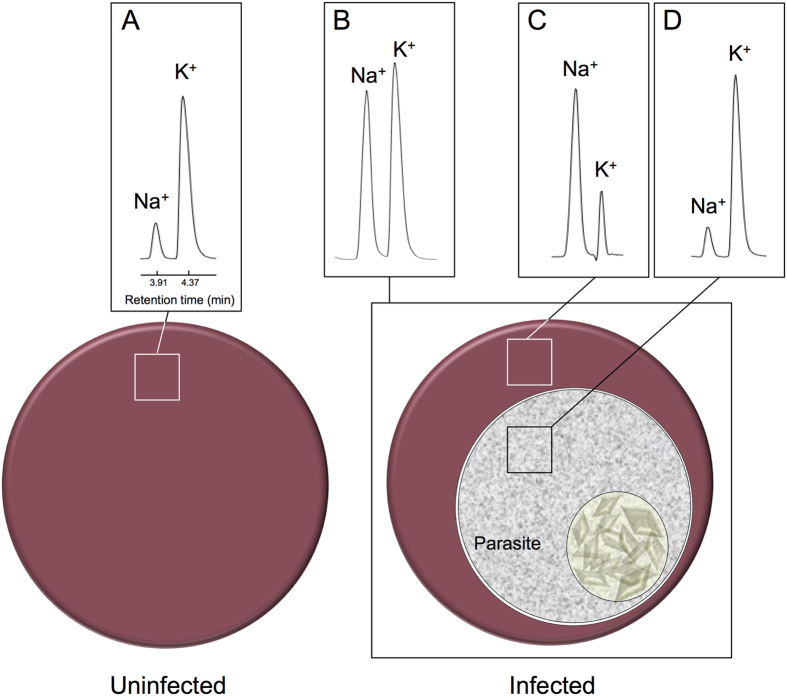
Na^+^ and K^+^ content of uninfected and *P. falciparum*-infected erythrocytes. Representative HPLC traces showing the Na^+^ and K^+^ content of (**A**) uninfected erythrocytes, (**B**) the *P. falciparum*-infected erythrocyte, (**C**) the cytosol of the infected erythrocyte (obtained by saponin-permeabilisation of the infected erythrocyte membrane, with subtraction of a saponin ‘blank’ to account for the Na^+^ and K^+^ content of the saponin stock), and (**D**) the saponin-isolated parasite.

In initial experiments the method was applied to uninfected human erythrocytes, yielding a Na^+^ content of 6.2 ± 0.3 mmol/10^13^ cells and a K^+^ content of 99 ± 5 mmol/10^13^ cells (n = 6, mean ± SD) (Fig. 1A), consistent with well-established literature values^12^. Application of the method to human erythrocytes infected with the mature asexual trophozoite form of the malaria parasite, *Plasmodium falciparum* (approximately 36 h post-invasion), confirmed the long-recognised^13^,^16^,^17^ changes in the Na^+^ and K^+^ content of the infected cells, with Na^+^ estimated here to have increased to 52.6 ± 2.8 mmol/10^13^ cells (n = 6, mean ± SD, P < 0.01, paired t-test) and K^+^ estimated here to have decreased to 70 ± 4 mmol/10^13^ cells (n = 6, mean ± SD, P = 0.01, paired t-test) (Fig. 1B).

Analysis of the Na^+^ and K^+^ contents of the parasite and host cell compartments (separated from one another through the selective release of the contents of the host cell compartment by permeabilisation of the erythrocyte membrane with saponin) confirmed that, as recognised previously^14^,^16^,^17^,^18^,^19^, the net changes in the Na^+^ and K^+^ content of the parasite-infected erythrocyte are attributable to a profound change in the Na^+^ and K^+^ concentrations in the host cell cytosol. In the uninfected erythrocyte the Na^+^/K^+^ ratio estimate was 0.06; in the infected erythrocyte cytosol Na^+^ increased to 52.3 ± 1.2 and K^+^ decreased to 13.2 ± 1.9 mmol/10^13^ cells, a Na^+^/K^+^ ratio of 3.9 (n = 6, mean ± SD; Fig. 1C). The intracellular parasite itself maintains Na^+^ and K^+^ levels of 2.1 ± 0.1 and 41.7 ± 2.2 mmol/10^13^ cells, respectively, a Na^+^/K^+^ ratio of 0.05 (n = 6, mean ± SD; Fig. 1D), similar to that in the uninfected erythrocyte. The cell water volume of the mature asexual trophozoite *P. falciparum* parasite has been estimated as 28 fl^20^. If it is assumed that the cell Na^+^ and K^+^ is distributed throughout the intracellular water volume this equates with intracellular concentrations of 7.5 mM for Na^+^ (close to the value of 11 mM estimated previously for isolated 3D7 parasites using the fluorescent Na^+^-sensitive dye SBFI 7) and 149 mM for K^+^.

**Figure 2:**
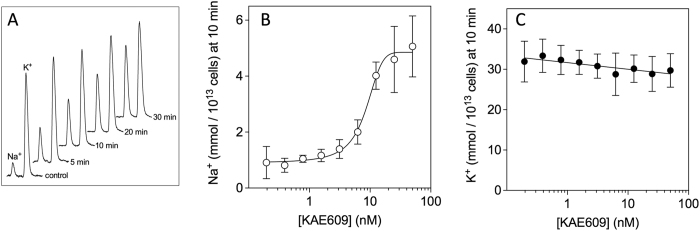
Effect of KAE609 on the Na^+^ and K^+^ content of saponin-isolated P. *falciparum* parasites. (**A**) HPLC Traces (representative of those obtained in 3 independent experiments) showing the time-dependent change in Na^+^ and K^+^ content of saponin-isolated parasites (10^6^ cells for each time point) over 30 min following the addition of 50 nM NITD609. (**B**,**C**) show the concentration dependence for the effect of KAE609 on (**B**) Na^+^ and (**C**) K^+^ as measured 10 min after the addition of KAE609. The data are averaged from nine independent experiments conducted on five different days and are shown ± SD. The Na^+^ data are fitted by a non-linear sigmoidal function and the K^+^ data by a straight line.

The utility of the HPLC method described here for screening compounds for their ability to disrupt transmembrane ion gradients was assessed using the clinical spiroindolone antimalarial candidate KAE609. The compound was added to saponin-isolated parasites at a concentration of 50 nM and the Na^+^ and K^+^ content of the parasites was monitored by HPLC. As can be seen in Fig. 2A, on addition of KAE609 there was a progressive time-dependent rise in parasite Na^+^ content, increasing 13-fold to 31 ± 4 mmol/10^13^ cells after 30 min (n = 3, mean ± SD, P = 0.004, paired t-test). Over the same time period the K^+^ content of the parasites appeared to decrease slightly; however, the small decrease in K^+^ evident after 30 min was not statistically significant (n = 3, mean ± SD, P = 0.1, paired t-test).

The method was applied to assessing the effect of four other spiroindolones on the Na^+^ and K^+^ content of the parasites. NITD246 and NITD139 are potent inhibitors of parasite growth (NITD246 moreso than NITD139) whereas NITD247 and NITD138 are, respectively, their largely inactive enantiomers (both have residual activity, most likely due to the presence of trace amounts of the active enantiomer in each)^7^. As shown in Table 1, the addition of NITD246 and NITD139 (and to a much lesser extent NITD247), each at 50 nM, gave rise to a significant increase in parasite Na^+^ content as measured after 30 min, with the order of the magnitude of the increase (NITD246 > NITD139 ≫ NITD247 > NITD138) matching that of the order of their potency in inhibiting parasite growth (Table 1) and the order of their potency in inducing a rise in the cytosolic [Na^+^] as estimated previously using the fluorescent Na^+^ indicator SBFI 7. The HPLC method is therefore able to distinguish between compounds of differing potency.

None of these other spiroindolones had a significant effect on parasite K^+^ content as measured after 30 min exposure (not shown).

The concentration-dependence of the effect of KAE609 on parasite Na^+^ and K^+^ content was assessed by adapting the technique to a higher-throughput 96-well plate format. Figure 2(B,C) show dose-response curves for the effect of KAE609 on parasite Na^+^ and K^+^ content, as measured 10 min after the addition of the compound. The EC_50_ (i.e. the concentration at which the effect of KAE609 on parasite Na^+^ content was half maximal) was estimated as 7.9 ± 1.5 nM (mean ± SD; n = 9; Fig. 2B). The small effect of KAE609 on parasite K^+^ content as measured at 10 min, over the range of KAE609 concentrations tested here, was not statistically significant (P = 0.08, two-way ANOVA).

In summary, the HPLC method described here allowed the high-sensitivity determination of the Na^+^ and K^+^ content of the parasite and host cell compartments of the malaria-parasite infected erythrocyte. The method required very small volumes of cells and was readily adapted to a 96-well plate format. The method is readily applied to a broad range of cell-types and may be adapted for use with other ions^21^,^22^. As such, it provides a ready means for the high throughput screening of compounds for their ability to disrupt cell ion homeostasis.

The method offers particular advantages relative to other methods presently available for assessing cell ion composition. It combines simplicity of sample preparation, high-sensitivity, multi-ion detection and adaptability to a high-throughput multi-well plate format. Alternative analytical techniques applied to study Na^+^/K^+^ homeostasis in uninfected and malaria parasite infected erythrocytes in most cases deliver a comparable sensitivity, with an LOD/LOQ (Limit of Detection/Limit of Quantification) in the low μM range, but have some disadvantages relative to the methodology described here. X-ray microanalysis^17^,^18^ allows multi-ion detection but requires expensive equipment and extensive sample preparation and is not suited to a high throughput format. Ion-sensitive electrodes^14^ or microelectrodes^15^ are comparatively cheap and are able to measure ion concentrations in the low μM range^23^; however, they can be susceptible to cross-interference from other ions and require a different electrode for each analyte^23^. Flame photometry and atomic absorption spectrometry have long been applied to the quantitation of Na^+^ and K^+^ in biological samples^12^,^13^ and have been used for the determination of the Na^+^ and K^+^ content of both uninfected^12^ and infected^16^ erythrocytes, but require relatively large sample volumes and are not suited to high throughput measurements. Fluorescent ion-indicators such as the Na^+^-sensitive dye SBFI^7^,^8^,^9^,^10^,^11^ provide the opportunity to monitor and quantify intracellular ion concentrations *in situ*, but require intact and viable cell preparations, are applicable to single ion species within a relatively narrow dynamic range, and are available for a very limited number of ions.

## Methods

### Cells and spiroindolones

*P. falciparum*-infected red blood cells (iRBCs; infected with 3D7 parasites), were cultured under standard conditions^24^. Synchronised trophozoite-stage iRBCs, approximately 36 h post-invasion, were enriched to a parasitaemia of 98–99%, using a VarioMACS (Miltenyi Biotech) as described elsewhere^25^. Uninfected red blood cells (RBCs) were incubated under the same conditions as iRBCs for 36 h prior to experimentation.

KAE609 was obtained from the Medicines for Malaria Venture. NITD246, NITD247, NITD 138 and NITD139 were generously provided by the Novartis Institute for Tropical Diseases (Singapore).

### HPLC

HPLC was conducted on a Dionex Ultimate 3000 RSLC with a Corona Ultra Charged Aerosol Detector (Dionex). The detector was set to a gain range of 20 pA, with a 60 Hz data collection rate. The nebuliser was set to 35 °C. For all analyses other than those of samples generated using the 96-well plate format the instrument was fitted with an Acclaim Trinity P1 2.1 × 150 mm, 3 μm column (Thermo Fisher Scientific) operated at 35 °C. The mobile phase was 40/60% (v/v) 20 mM ammonium acetate pH 5/Acetonitrile; the flow rate was 0.5 mL/min^21^,^25^ and the run time 15 min.

### Analysis of intracellular Na^+^ and K^+^ content

To determine the intracellular Na^+^ and K^+^ content of RBCs and iRBCs, an aliquot of approximately 10^7^ cells was washed twice (16,000 × g, 10 s) in 1 mL of ice-cold ‘erythrocyte wash solution’ (107 mM Magnesium acetate in HPLC-grade water, adjusted to 320 mOsm and pH 7.4). The washed cells were then lysed by the addition of 100 μL of a ‘lysis solution’ (40%/60% (v/v) 20 mM Ammonium acetate pH 5/Acetonitrile). The lysate was centrifuged at 16,000 × g for 5 min to remove debris and 10 μL of the resulting supernatant, solution, containing the intracellular ions, was transferred to a vial for HPLC analysis.

To determine the Na^+^ and K^+^ content of the infected red blood cell cytosol and that of the intracellular parasite, approximately 10^7^ enriched iRBCs (≥98% parasitaemia) were incubated for 5 min in 100 μL of ice-cold ‘erythrocyte wash solution’ containing 0.05% w/v saponin (of which≥10% was the active agent sapogenin). The saponin permeabilises the infected erythrocyte membrane, thereby releasing the iRBC cytosolic contents into the (Na^+^- and K^+^-free) medium whilst maintaining the integrity of the parasite plasma membrane^20^,^26^. At the end of the 5 min incubation the sample was centrifuged at 16,000 × g for 1 min to sediment the ‘isolated’ parasites; the supernatant solution, containing the iRBC cytosol contents, was transferred to a new microfuge tube then centrifuged at 16,000 × g for 5 min to remove cell debris. A 25 μL aliquot of the supernatant solution was mixed with 75 μL lysis solution and 10 μL of the resulting solution was analysed by HPLC. The isolated parasites were resuspended and washed twice in ice-cold ‘parasite wash solution’ (107 mM magnesium acetate in HPLC-grade water, adjusted to 320 mOsm and pH 7.1) and the cell pellet was then lysed by the addition of 100 μL of lysis solution. The lysate was centrifuged at 16,000 × g for 5 min to remove debris and 10 μL of the resulting supernatant solution was analysed by HPLC.

Early experiments revealed trace levels of Na^+^ and K^+^ originating from the saponin stock, necessitating a correction being applied to the Na^+^ and K^+^ levels measured in the diluted red blood cell cytosol samples in which the added saponin solution remained present. In the case of the isolated parasite samples, the multiple washes of the parasites in the (Na^+^- and K^+^-free) parasite wash solution removed the (saponin-derived) residual Na^+^ and K^+^; there was therefore no correction necessary for these samples. To quantify the Na^+^ and K^+^ content of the saponin stock, a 25 μL aliquot of a 0.05% w/v saponin solution in erythrocyte wash solution was mixed with 75 μL lysis solution and 10 μL of the resulting solution was analysed by HPLC. The Na^+^ and K^+^ content of the diluted saponin stock solution (2.5 ± 0.7 pmol/μL (n = 6, mean ± SD) and 82 ± 7 pmol/μL (n = 6, mean ± SD), respectively) was subtracted from that of the supernatant solution containing the iRBC cytosol contents (in which the average Na^+^ and K^+^ concentrations were 133 ± 3 pmol/μL (n = 6, mean ± SD) and 115 ± 5 pmol/μL (n = 6, mean ± SD), respectively).

### Effect of spiroindolones on parasite Na^+^ and K^+^

Parasites isolated as described above were resuspended at 37 °C in phosphate-buffered saline (PBS; Sigma Aldrich) pH 7.1 supplemented with 20 mM glucose. Spiroindolones were added at a concentration of 50 nM unless specified otherwise. Aliquots of the parasite suspension (typically, 200 μL) were taken at predetermined intervals and the parasites were washed twice with ice-cold parasite wash solution as described above, before being lysed by the addition of 100 μL of lysis solution. The lysate was centrifuged (16,000 × g, 5 min) to sediment debris and the Na^+^ and K^+^ content of the supernatant solution (10 μL) was analysed by HPLC.

### Adaptation to a 96-well plate format

The assay was adapted to a 96-well plate format using plates with wells having a V-shaped bottom (Corning). The V-shape geometry assisted in the formation of a cell pellet on centrifugation of the plate. Multichannel pipettes and a multichannel aspirator were used for transfer and wash steps. For analysis of the 96-well plate samples the HPLC instrument was operated in tandem mode using two Acclaim Trinity P1 3.0 × 50 mm columns (Thermo Fisher Scientific) on a 2-position 10-port switching valve and a dual-gradient pump (both Dionex). This setup significantly reduced the sample analysis time, from 15 min to 3.5 min per sample. Using this format, 96 wells (i.e. one plate) could be analysed in 5.6 h.

The 96-well plate format was used to obtain dose-response curves for the effect of KAE609 on parasite Na^+^ and K^+^ content. A serial dilution of KAE609 in PBS pH 7.1 containing 20 mM glucose (final volume 100 μL) was prepared in a 96-well plate with V-shaped wells. Saponin-isolated parasites suspended in PBS pH 7.1 containing 20 mM glucose (100 μL) were added, giving a total volume of 200 μL with parasite numbers ranging between experiments from 4 × 10^6^ to 5 × 10^7^ per well. The plate was incubated at 37 °C for 10 min, at the end of which the cells were separated from the extracellular solution by centrifugation of the plate (2000 × g for 1 min) and washed twice with ice-cold parasite wash solution. The cells were lysed in 100 μL lysis solution, and the plate was then centrifuged (2000 × g, 5 min) to sediment debris. The supernatant solutions were transferred to a new plate which was sealed using either an Aluminium foil seal (Diversified Biotech) or a well plate sealing mat (Thermo Fisher Scientific). A 10 μL aliquot of each sample was analysed by HPLC for Na^+^ and K^+^ content.

### Method Validation

The retention times for Na^+^ and K^+^ on the Acclaim Trinity P1 2.1 × 150 mm column (used for all analyses other than those involving the 96-well plate format) were 3.91 ± 0.03 min and 4.37 ± 0.05 min, respectively (n = 30, mean ± SD). The retention times for Na^+^ and K^+^ on the Acclaim Trinity P1 3.0 × 50 mm column (used for the analysis of the 96-well plate samples) were 1.89 ± 0.07 min for Na^+^ and 2.23 ± 0.08 min for K^+^ (n = 90, mean ± SD). The linearity and calibration range of the HPLC method was assessed using eight-point calibration curves for both Na^+^ and K^+^, dissolved as Cl^−^ salts in water. A power regression model with no weighting gave a mean correlation coefficient of 0.999 ± 0.001 for both analytes (n = 12). The LOD and LOQ were determined to be 5.6 ± 1.3 pmol and 39.2 ± 2.1 pmol, respectively, for Na^+^, and 7.3 ± 1.1 pmol and 51.4 ± 2.5 pmol, respectively, for K^+^. The selectivity of the method was evaluated by measuring the Na^+^ and K^+^ content of the lysis solution as well as that of the erythrocyte and parasite wash solutions. There were no interfering signals detected in these samples and none of the other ions present in these solutions co-eluted with the Na^+^ or K^+^. The precision of the method was determined by analysing three different quality control samples (0.1, 1 and 10 nmol of each of Na^+^ and K^+^) in independent assays, measured on different days (n = 4), revealing a total-assay variation of 1.5% (two-way ANOVA).

## Additional Information

**How to cite this article**: Winterberg, M. and Kirk, K. A high-sensitivity HPLC assay for measuring intracellular Na^+^ and K^+^ and its application to *Plasmodium falciparum* infected erythrocytes. *Sci. Rep.* **6**, 29241; doi: 10.1038/srep29241 (2016).

## Figures and Tables

**Table 1:** Effect of various spiroindolones on the Na^+^ content of saponin-isolated *P. falciparum* parasites.

Spiroindolone	IC_50_ for inhibition of parasite proliferation (nM)*	Na^+^ content at 0 min (mmol/10^13^ cells)	Na^+^ content at 30 min(mmol/10^13^ cells)	P
NITD246	0.12	2.3 ± 0.4 (n = 3)	38.6 ± 3.0 (n = 3)	0.001
NITD247	41.6	2.3 ± 0.2 (n = 3)	2.9 ± 0.2 (n = 3)	0.05
NITD138	2300	2.2 ± 0.1 (n = 3)	2.2 ± 0.2 (n = 3)	NS
NITD139	4.0	2.1 ± 0.1 (n = 3)	11.3 ± 2.0 (n = 3)	0.008

Each spiroindolone was added to a final concentration of 50 nM to saponin-isolated parasites suspended in PBS, pH 7.1, supplemented with 20 mM glucose, at 37 °C. The parasites were sampled for HPLC analysis immediately prior to (i.e. at 0 min), and 30 min after, the addition of spiroindolone. The P values, indicating the significance of the change in parasite Na^+^ content over the 30 min incubation period, were derived from paired t-tests; NS = Not Significant.

^*^IC_50_ values for inhibition of (3D7) parasite proliferation are taken from ref. 7.
